# Parental approaches to sexual and reproductive health information communication with adolescents in Ghana

**DOI:** 10.3389/fpubh.2025.1609491

**Published:** 2025-07-14

**Authors:** Frank B. Agyei, Doreen K. Kaura, Janet D. Bell

**Affiliations:** Department of Nursing and Midwifery, Faculty of Medicine and Health Sciences, Stellenbosch University, Stellenbosch, South Africa

**Keywords:** adolescent, parent, approach, sexual and reproductive health, information, communication, Ghana

## Abstract

**Background:**

Parent-adolescent sexual and reproductive health (SRH) communication is recognized as an important protective factor against risky sexual behaviors and associated health problems, such as sexually transmitted infections (STIs) and unintended pregnancies. This study explored the specific approaches Ghanaian parents use to communicate SRH information to their adolescents.

**Methods:**

This study employed a qualitative descriptive design to conduct interviews with 10 parents, with the sample size determined by data saturation. Participants were selected through purposive sampling from Asante Akyem North Municipality in Ghana. Thematic analysis was conducted inductively, following Braun and Clarke's approach.

**Findings:**

Two broad themes emerged: timing of sexual and reproductive health information communication and sexual and reproductive health information communication style. This study highlights varying parental approaches to sexual and reproductive health communication, ranging from proactive and open to authoritative communication.

**Conclusion:**

The findings underscore the complexity of parent-adolescent SRH communication, shaped by cultural norms, parental comfort levels, and perceived adolescent maturity. This highlights the need for interventions that enhance parent-adolescent communication skills.

## Introduction

Adolescents' sexual and reproductive health (SRH) plays a vital role in their overall wellbeing, especially in low- and middle-income countries where the burden of disease is significantly high (1). Communication about SRH between parents and their adolescents is widely recognized as an important protective factor against risky sexual behaviors and associated health problems, such as sexually transmitted infections (STIs) and unintended pregnancies (2, 3).

Globally, SRH communication is influenced by socio-cultural, religious, and familial norms. In particular, parents play an instrumental role in shaping their children's understanding of SRH issues, but the extent and nature of parental involvement vary across countries and cultures (4, 5). In Western contexts, studies have shown that parents tend to engage in relatively open and proactive SRH discussions with their children (6). However, in many parts of sub-Saharan Africa, including Ghana, SRH communication between parents and adolescents is often less direct and influenced by cultural taboos, religious beliefs, and fears surrounding adolescent sexual activity (7).

In sub-Saharan Africa, the sociocultural context adds complexity to how SRH information is conveyed within families. Traditionally, discussing matters related to sexuality has been considered taboo (8). Studies indicate that many parents in the region feel uncomfortable or unprepared to have such conversations with their children, often perceiving such discussions as inappropriate or morally compromising (9). This reluctance may lead to avoidance, delayed conversations, or reliance on indirect forms of communication such as the use of proverbs or stories to convey messages related to sexual behavior. For example, research conducted in Kenya by Maina et al. (10) found that only a small proportion of parents actively engaged in SRH discussions with their adolescent children, citing fears that such conversations might encourage early sexual activity. Similar trends have been reported in Ghana, where parental discomfort and concerns about cultural expectations shape communication patterns (11). Consequently, adolescents in these contexts may rely on peers or external sources of information, often receiving incomplete or inaccurate SRH information, which can put them at a higher risk of poor SRH outcomes (12).

Furthermore, communication styles vary between the urban and rural settings. In rural areas, where traditional values are more strongly upheld, parents are less likely to openly discuss SRH with their children (13). Parents in urban settings may be more likely to provide some form of SRH education, but these discussions are often reactive and occur after parents learn that their adolescent child has begun engaging in sexual activities, as opposed to proactively before such behaviors arise (13).

One notable issue affecting SRH communication in Ghana is the lack of formal training or resources for parents to feel equipped to discuss these topics. Studies have shown that many parent in Ghana feel ill-prepared to have conversations about sexual health, often lacking the correct information themselves (14). This creates a significant gap, as adolescents may be left to seek SRH information from less reliable sources, such as their peers or the Internet, increasing their risk of engaging in unsafe sexual behaviors.

The rationale behind this study stems from the growing need to address adolescent SRH issues in Ghana, particularly in light of rising concerns about adolescent pregnancy rates and the spread of STIs, including HIV (15). Despite these public health concerns, there is limited research on how parents approach SRH communication, particularly in culturally nuanced contexts. Most existing studies focus on barriers to communication, such as parental discomfort or cultural taboos, without delving deeply into the strategies parents employ or how these strategies impact adolescent health outcomes.

Given the critical role that parents play in shaping adolescents' sexual behavior, understanding the diverse approaches to SRH communication within the Ghanaian context is essential. While SRH education in schools and among health professionals is important, parental influence is often more direct and deeply embedded in adolescents' decision-making processes. Therefore, this study aimed to explore the specific approaches that Ghanaian parents use to communicate SRH information to their adolescents.

## Materials and methods

### Study design

This is the second phase of an explanatory sequential mixed method study. In the first phase, a systematic review was conducted to identify effective interventions to improve SRH information communication in LMICs (2).

This study used a qualitative descriptive approach (16, 17). This approach is commonly applied in qualitative research that aims to provide a descriptive account, particularly in studies that focus on health-related issues. This is grounded in the fundamental principles of naturalistic inquiry, allowing researchers to capture participants' experiences in their own words (16). This design ensures that the data analysis remains true from the participants' perspectives, enhancing the transparency of the researcher's descriptions (17).

### Study setting

This study was conducted in the Asante Akyem North Municipality of Ghana, a region characterized by diverse cultural and religious backgrounds. This diversity makes it a suitable location for exploring the various approaches parents use to communicate SRH information to their adolescents. The main languages spoken in the municipality are English and “Asante Twi,” with additional ethnic languages in use. Language not only serves as a cultural marker, but also plays a crucial role in preserving and transmitting cultural values and norms.

### Participants, sampling, and sample size

Parents of adolescent children were recruited for the study. They included biological mothers or fathers, as well as male or female guardians of adolescents aged 13–16 years who were willing to participate. Parents of adolescents within the stated age range were selected through purposive sampling, ensuring that they were included based on the objectives of the study. To ensure variability amongst parents, a minimum of three males and three females were sampled. Parents were biological mother or father or a male/female caretaker. Recruitment materials such as informational flyers were distributed to potential participants at the community center. Consent was obtained prior to participation.

To capture developmental variations among adolescents, the study included five parents of adolescents aged 13 to 14 years and five parents of adolescents aged 15 to 16 years. To ensure diversity in perspective, at least three male and three female parents were included in the sample.

The final sample consisted of 10 parents, as determined by data saturation. Saturation was reached after analyzing data from the eighth parent, as no new themes or codes emerged. To confirm this, two additional interviews were conducted to reinforce the consistency of the identified themes. The research team met to review coding patterns and agreed that sufficient depth and variation had been achieved to address the research questions. This methodological approach aligns with established qualitative research principles, which define saturation as the point at which further data collection no longer contributes novel insights or findings (18).

### Data collection

Individual interviews were conducted using a semi-structured interview guide. This guide was developed by the research team, drawing on findings from a systematic review conducted in the first phase of the study. The guide included open-ended questions with probes and prompts to facilitate the collection of in-depth and contextually rich data from parents.

The guide was developed in English as that is the national language in Ghana and is widely used. This notwithstanding, another version was developed in Asante Twi, which is the predominant local language in the study setting. This was translated back into Asante Twi to ensure the translation was valid. The interview guide begins with the question: “let us talk about your communication with your adolescent.” The interviewers used probes and active listening skills, reflections and summarizing all through the interviews and also noted non-verbal cues from both parents and adolescents.

A pilot interview was conducted to assess the clarity of the questions and estimate the interview duration. The collected data were transcribed, analyzed, and reviewed by the supervisors. Based on the feedback, minor adjustments were made to the interview guide to ensure that parents' experiences with SRH communication with their adolescents were thoroughly explored.

Written informed consent was obtained from participants before the interviews. Interviews were scheduled at a time and location that was convenient for the participants. Participants preferred to be interviewed in their home environment, and this was done accordingly.

Face-to-face semi-structured interviews were conducted by the researcher and trained research assistants with experience in qualitative interviews. All interviews were audio-recorded with participants' permission. Only the researcher, research assistants, and interviewees were present during the sessions. Interviews were conducted in English and “Asante Twi” languages.

Each interview lasted between 35 and 60 min. The data collection for this phase of the study was conducted between August 2022 and January 2023.

### Data analysis

Data analysis was conducted inductively using Braun and Clarke's thematic analysis approach (19). The doctoral student first listened to the recorded interviews multiple times, both before and after the transcription, to gain familiarity with the data. The interviews were transcribed verbatim in either Asante Twi or English. Those conducted in Asante Twi were first transcribed, translated into English, and subsequently back-translated into Asante Twi for accuracy, ensuring that the participants' perspectives were faithfully represented.

Following the transcription, the doctoral student cross-checked the transcripts against the original recordings to verify accuracy. Although direct participant verification of the English translations was not possible, a rigorous multistep translation and verification process was implemented to maintain data integrity. The verified transcripts were then uploaded to Atlas.ti (version 23.0.7) for systematic organization into meaningful units, leading to the development of initial codes. Coding was done by the researcher, and the research assistant also did for crosschecking. The supervisors also crosschecked these to ensure credibility.

These codes were categorized and refined into themes and subthemes to ensure minimal overlap. The final themes were aligned with the study objectives, clearly defined, and named. A comprehensive report was generated based on these findings.

### Trustworthiness

Trustworthiness was maintained throughout the study to ensure an accurate representation of participants' experiences. This study employed credibility, confirmability, transferability, and dependability as key measures of trustworthiness (20). To achieve this, strategies, such as the use of probes and prompts, follow-up interviews, and member checking which included validation of themes, were implemented.

### Ethics

This study complied with the ethical principles outlined in the Declaration of Helsinki, which established the guidelines for conducting research involving human participants (21). Ethical approval was obtained from the Health Research Ethics Committee (HREC) of Stellenbosch University (reference number S21/08/159) on January 6, 2022. Additionally, the approval was renewed and extended on January 6, 2023.

Following HREC approval, ethical clearance was secured in Ghana from the Committee on Human Research, Publication, and Ethics (CHRPE) of the College of Health Sciences at Kwame Nkrumah University of Science and Technology. This approval was granted on July 19, 2022, with reference number CHRPE/AP/356/22. Those who were interested in taking part of the study signed a written consent form.

## Results

### Characteristics of parents

Ten parents of adolescents took part in the study. Four of them were male and six were female parents. Other participant characteristics are detailed on Table 1.

**Table 1:** Participants demographic characteristics.

**Participant**	**Gender**	**Level of education**	**Tribe**	**Language**	**Religion**
Simpoa	F	Primary	Ashanti	Asante Twi	Christianity
Kyirekua	F	No formal education	Frafra	Frafra, Asante Twi	Islamic
Aku	F	No formal education	Ashanti	Asante Twi	Christianity
Dua	M	Tertiary	Akuapem	English, Akuapem Twi	Christianity
Sisi	F	Tertiary	Ashanti	English, Twi	Christianity
Brakatu	F	Tertiary	Ashanti	English, Twi	Christianity
Sika	M	Tertiary	Fanti	English, Fante Twi	Christianity
Akyaw	M	Tertiary	Ashanti	English, Asante Twi	Christianity
Otuo	M	Tertiary	Anlo Ewe	English	Christianity
Kudi	F	Tertiary	Dagomba	English, Twi, Haussa	Islamic

This study explored various approaches used by parents in Ghana to communicate SRH issues with their adolescents. Two key themes emerged from the analysis, each with its corresponding subthemes. These themes include the Timing of SRH Information Communication and SRH Information Communication Style. These themes, along with their subthemes, are summarized in Table 2.

**Table 2:** Themes and subthemes.

**Themes**	**Subthemes**
Timing of SRH information communication	• Proactively initiating SRH discussion • Reactively addressing SRH issues • Age specific discussions • Progressive introduction of topics
SRH information communication style	• Open communication • Subtle communication • Authoritative

### Timing of SRH information communication

This theme refers to when parents choose to discuss sexual SRH issues with their adolescents. It captures the factors influencing the timing of these discussions and the stages in adolescents' lives when they occur. The subthemes included proactively initiating SRH discussions, reactively addressing SRH issues in response to adolescents' questions, and age-specific discussions.

#### Proactively initiating SRH discussion

This subtheme refers to parents consciously initiating SRH discussions before adolescents are externally exposed to these issues. It captures the parent's purposeful strategy to open SRH discussions in a relatable and approachable manner.

Some parents highlighted the importance of sharing SRH information with adolescents as early as possible to help them obtain the correct information before they contact their peers. One shared that:

“*I think we have to start it earlier. We don't have to wait for them to reach that stage because some kids will not have confidence in their parents and as they reach that stage, they will rather go to their friends and peers. So, if you start it earlier with the kid, he will become, he will become, how do I say it, free or …….. he won't feel shy to tell you anything*.”    Sisi, F

According to the participant, by fostering this early bond, the adolescent is more likely to approach the parent for guidance rather than rely on peers, who may provide incomplete or inaccurate information.

Some participants highlighted their deliberate efforts to introduce sensitive SRH topics in ways that would resonate with adolescents. One parent shared:

“*You know because I've got good relationship with them, introducing these topics are deliberately, so what happens is that before introducing these topics…. I have already psyched myself how I'm going to do it as at times it is introduced in a form of joke so it comes to me naturally”*    Otuo, M

This narrative illustrates the parents' intentional and thoughtful approach to creating an open environment for SRH discussions. By leveraging a strong parent-adolescent relationship and incorporating humor, they actively facilitated conversations in a manner that felt organic and comfortable.

#### Reactively addressing SRH issues

This subtheme refers to parents waiting until an event or situation prompts the need for SRH discussion. Some parents waited until the adolescents started developing secondary sexual characteristics. Some shared that:

“*For me…. I will not say a specific age, but I will say that a parent if you see your girl child developing girl features like the breasts, the armpits and the private part started to grow hairs, then it means you have to start*.”    Dua, M

“*Because… the menses hasn't started, I wasn't speaking it with her, but when it started, I started introducing it to her talking to her verbal, so we did that*.”    Brakatu, F

These quotes highlight a reactive approach to SRH communication, where parents initiate discussions only after significant milestones such as the onset of menstruation. The reliance on biological markers and hesitation in phrasing suggests uncertainty or a lack of preparedness in addressing these topics proactively. This approach may delay important conversations, potentially leaving adolescents unprepared for the changes and challenges that they encounter.

#### Age specific discussions

This subtheme refers to the parents' timing of SRH communication based on the perceived maturity or developmental stage of the adolescent. Some parents mentioned that they want adolescents to reach a certain level of perceived maturity before they share certain SRH information with them. Thus, the type of information depends on the maturity level of the adolescent. Some expressed that:

“*Initially when they were growing up, I tried as much as possible to hide those sexual things from them because I think that time they were not prepared to be exposed to such adolescent conversations but when they started reaching adulthood*.”    Otuo, M

“*Errm… possibly at the secondary school especially the junior secondary, so at least at the secondary school level, where she will be more matured, I can upgrade the conversation better as she prepares for tertiary education where the trends and other things will be more, the threats, the temptations and the desire will be more…so from the secondary school, I will upgrade the conversation.”*    Akyaw, M

These quotes highlight a cautious, staged approach to SRH communication, in which parents delay discussions until they perceive that the adolescent is mature enough. Early conversations may be avoided to “protect” younger children, with deeper topics introduced during adolescence, particularly at the secondary school level, as perceived risks and temptations increase. Parental experiences and developmental milestones significantly influenced the timing and content of these discussions.

#### Progressive introduction of topics

This theme reflects an incremental and empathetic method in which parents tackle sensitive SRH topics in stages, allowing both themselves and their adolescents to adjust to the emotional and cultural complexities of the conversation over time.

In some cultural contexts, discussing topics related to sexuality can be challenging because of societal norms and discomfort associated with such conversations. A parent recounted their experience navigating this sensitive terrain, sharing how initial discussions about the menstrual cycle were met with ease, but transitioning to topics about sexual activities brought about visible discomfort to their adolescents.

“*Eerm…. on our part of our world, it is difficult to talk about sexuality so when I started, she was comfortable at first with the menstrual cycle activities and all those things but when the sexual activities came in, she was very uncomfortable so I had to even break, go and come back later, and that's how it went. Even for me, it was somehow difficult but I needed to do it.”*    Sika, M

Recognizing the unease, the parents adopted a gradual approach, pausing the conversation, and revisiting the subject later. They acknowledged their own discomfort but emphasized the importance of addressing these topics, ensuring that the adolescents received the necessary information, even when the conversation was difficult for both parties.

### SRH information communication style

This theme pertains to how parents deliver SRH information to their adolescents. This theme encompasses the tone, approach, and overall style used by parents during SRH discussions, reflecting the varying levels of openness, authority, and engagement.

#### Open communication

This subtheme refers to how parents adopt a straightforward and clear style and openly discuss SRH issues without ambiguity. Parents encouraged adolescents to ask questions and engage freely, creating an environment in which parents could share their knowledge and provide guidance.

Some parents emphasized the informal, relaxed nature of their SRH communication with their adolescents. This approach is marked by openness and a lack of rigidity, which makes the conversation feel natural and non-threatening. One shared that:

“*We are free, we talk, we laugh, so it is like normal conversation. It is not something strict, I don't sit her down like strict, no no no no, we just communicate as we have been doing always*.”    Sisi, F

The parent reflects that they do not resort to formal or structured conversations when discussing sensitive topics, such as SRH, but instead maintain an easygoing and open dialogue. This style of communication promotes comfort and ease, allowing adolescents to feel more at ease when discussing difficult topics. The absence of strictness or authority figures in the conversation fosters a more open and honest exchange, highlighting the importance of establishing a trusting and approachable environment for discussing SRH matters.

Another parent shared that:

“*It doesn't look like I am teaching them something. They just bring their ideas and I also chip in mine*.”    Otuo, M

This illustrates a collaborative approach to SRH communication in which the parent views the conversation as a mutual exchange rather than a one-sided teaching experience. The emphasis is on creating an open, two-way dialogue where the adolescents feel comfortable sharing their ideas and thoughts. The parent contributes their perspective in a way that does not come across as formal instruction, but rather as part of an ongoing, open discussion.

#### Indirect communication

This subtheme involves parents conveying SRH information in a subtle and less explicit manner. Parents use euphemisms, analogies, or non-verbal cues to reflect an approach that may align with cultural norms, personal discomfort, or the desire to ease adolescents into sensitive subjects.

“*So I use sometimes scenarios and things that I come across, experiences to explain things to her. Because she is my first born, so I re-echo it so that she doesn't forget*.”    Brakatu, F

Some parents communicated with their adolescents by using proverbs. They relied on this approach to provide information on SRH to their adolescents. One shared that:

“*I sometimes use proverbs when speaking to my child. If you speak too straight about it, you are seen to be a bad person*.”    Akyaw, M

Using scenarios, experiences, and proverbs to explain topics reflects an indirect approach because the parent does not explicitly address the issue, but instead uses illustrative examples to convey the message. This strategy may allow sensitive topics to be discussed subtly, which may make the conversation more comfortable for parents and adolescents.

#### Authoritative communication

This is about how parents provided structured guidance, set firm boundaries, and focused on clear rules to protect adolescents.

In many traditional settings, parents emphasize protective and cautionary advice to guide adolescents through sensitive stages of life, particularly during puberty. A parent shared how their own upbringing was shaped by strict teachings from their mothers, such as avoiding friendships and staying away from boys during menstruation, to prevent unwanted pregnancies and school dropouts. This approach focuses on preserving societal expectations and safeguarding adolescents' reputations.

“*Don't make friends, and when you menstruate and a guy calls you don't go. If you go, the consequence of it is pregnancy then you will drop out from school. After school come home.”*    Kyirekua, F

Similarly, another parent conveyed how they advised their daughter to be mindful of boys and maintain good hygiene during menstruation to avoid any form of disgrace. These narratives underscore a protective, authoritative style of communication, in which guidance is grounded in warnings and preventative measures aimed at shielding adolescents from perceived risks and societal judgment.

“*She should be careful with guys and when she menstruates, she should keep herself well so that she doesn't disgrace herself. That's what I've been advising her.”*    Aku, F

In summary, the findings revealed diverse approaches to SRH communication between parents and adolescents. Communication styles ranged from authoritative and protective to direct and open, with parents sometimes emphasizing cautionary advice to safeguard adolescents from societal risks, such as teenage pregnancy and school dropouts. Some parents preferred indirect communication, using scenarios, personal experiences, or humor to introduce sensitive topics, while others adopted a gradual approach, addressing more sensitive issues over time, such as sexual activities, as comfort levels improved. The dynamics of SRH communication are influenced by the quality of parent-adolescent relationships. Parents with closer bonds facilitated open and natural conversations, whereas in more conservative settings, topics such as sexuality were challenging to address, requiring effort and persistence to navigate cultural discomfort.

## Discussion

This study explores parental approaches to SRH information communication with adolescents in Ghana. These findings provide valuable insights into the timing, style, and dynamics of this communication.

### Timing of SRH information communication

This study revealed that timing plays a pivotal role in shaping SRH communication between parents and adolescents. It emphasizes various factors that determine when and how SRH topics are introduced, including proactive initiation, reactive responses to adolescent development, age-specific discussions, and progressively introducing topics. As it emerged in this study, proactively initiating SRH discussions was particularly emphasized by parents who believed in addressing these topics early, aiming to build trust and confidence in their adolescents. Early communication about sexual health promotes a sense of security in adolescents, reducing their likelihood of seeking information from unreliable sources (22). One study found that although parents prefer to be proactive and decide to start these conversations early, they also consider factors such as adolescents' age, physical maturity, or the perceived risk of engaging in sexual activity (23). Such reactive approaches, where parents waited until significant milestones such as menstruation, also emerged in the current study. These parents emphasized waiting for physical signs of sexual maturation before engaging in SRH discussions. This is consistent with Chandra-Mouli and Patel (24), who found that parents wait for physical signs of maturation or expect their adolescents to initiate conversations. This similarity may be attributed to the influence of socio-cultural norms in many low- and middle-income settings, where discussing sexuality is considered sensitive or even taboo until a child shows physical signs of puberty. In such settings, parents may view early discussions as inappropriate or fear that initiating SRH conversations early could encourage sexual experimentation. In addition, a qualitative review focusing on sub-Saharan Africa highlighted that parents often delay SRH discussions until they perceive that their children are mature enough to understand, typically around puberty (25). While this approach may feel more comfortable for parents, it may miss the opportunity to influence adolescents' behaviors and leave adolescents ill-prepared for the psychological and social challenges they face during puberty (24).

Furthermore, age-specific discussions reflect the importance of perceived maturity in determining the timing and depth of SRH communication. For example, some parents delayed discussions until their children reached secondary school, viewing this as an appropriate stage for in-depth conversations. This gradual approach is in line with the literature on staged SRH communication, as found by Mumah et al. (26) in Nairobi, and in Nigeria by Aliyu and Aransiola (27). The consistency between these findings and the current study likely reflects shared cultural values across sub-Saharan African contexts, where age and schooling milestones are viewed as proxies for readiness and maturity.

Another critical finding was the gradual introduction of SRH topics, particularly sensitive issues. Many parents shared their experiences of introducing topics such as menstruation with ease, but transitioning to discussions about sexual behavior was more challenging and they had to postpone such discussions. Similarly, a study conducted in Harar, Eastern Ethiopia, investigated factors influencing parent-adolescent SRH communication. The research found that parents often initiated discussions on less sensitive topics but struggled to address issues related to sexual behavior. This challenge is linked to sociocultural norms and a lack of adequate knowledge, resulting in incomplete or postponed conversations (28). The similarity in findings may be explained by the strong influence of sociocultural norms across many African contexts, where open discussions about sex are often considered taboo, especially between parents and children. In such settings, sexuality is typically surrounded by silence and moralistic overtones, which makes it difficult for parents to discuss sexual behavior explicitly.

The use of humor and relatability in introducing difficult topics is noteworthy. Parents used familiar experiences and scenarios to facilitate discussions. This aligns with a study that explored the role of humor in mother-adolescent sexual communication and found that incorporating humor encouraged mothers to engage more in discussions about sex with their children (29). This study indicated that humor could enhance attitudes, social norms, and self-efficacy regarding sexual communication, ultimately making these conversations more effective.

### SRH information communication style

The study also revealed that the style of SRH communication varies greatly, depending on parental preferences and cultural factors. Three main styles were identified: open, indirect, and authoritative communication.

Some parents in this study communicated openly with their adolescents on SRH issues, which were fostered by good parent-adolescent relationships. In Uganda, Ndugga et al. (30) found that good parent-adolescent relationship is a facilitator of open parent–adolescent SRH information communication. The literature underscores that open SRH information communication is important for reducing risky sexual behaviors among adolescents (31). However, such discussions are often controlled by factors, such as parental knowledge deficits, cultural norms, and discomfort (32, 33). Some parents are concerned that talking about SRH may encourage sexual activity and hold the perception that adolescents are too young to grasp information (33). Despite these issues, both parents and adolescents acknowledge the significance of engaging in SRH conversations (31, 34).

In the current study, it was found that some parents preferred to deliver SRH information indirectly, using metaphors or euphemisms to soften the conversation. Similarly, other studies have found that certain parents opt for indirect methods when discussing SRH, often incorporating metaphors, euphemisms, and innuendos to make conversations more approachable (35, 36). Indirect communication, which is common in many cultures, may create barriers to understanding and lead to misinterpretation. This approach is especially common in conservative societies, where efforts are made to overcome the stigma associated with SRH discussions (37). This indirectness, although culturally grounded, may result in confusion, as adolescents may struggle to grasp the full meaning of these metaphors, potentially hindering their ability to make informed decisions.

The authoritative style, characterized by parents setting clear boundaries and providing guidance, was also evident in this study. For instance, some parents offered cautionary advice to their daughters regarding interactions with boys during menstruation, emphasizing societal expectations and pregnancy risks. This approach resonates with the findings of Pichon et al. (38) in central Uganda, where parents had strict SRH communication with adolescents. The same approach was found in Kenya by Maina et al. (10) who investigated parent-child SRH communication among very young adolescents in Korogocho. The similarity in findings across these studies can be attributed to the shared cultural norms, societal expectations, and gendered perceptions of adolescent sexuality in many African contexts.

## Limitations

While the findings offer valuable theoretical and practical insights into adapting a culturally appropriate intervention for SRH information communication, certain limitations should be considered when interpreting the results.

A significant proportion of the participants had attained tertiary education, which may have shaped the findings. Therefore, caution should be exercised when generalizing the results to a broader population. Furthermore, the study focused on parents of adolescents aged 13–16 years, meaning that the communication approaches of parents with older adolescents were not captured. While it is possible that similar experiences occurred when those adolescents were within the study's age range, generalizing the findings beyond this group should be done with caution.

## Conclusion and recommendation

This study highlights the diverse approaches that parents use to communicate SRH information to adolescents in Ghana. While proactive and open strategies promote effective communication, reactive, and authoritative approaches may limit adolescent preparedness. Interventions targeting parental engagement should consider cultural contexts and emphasize early, open, and progressive communication strategies to improve SRH outcomes in adolescents.

## Data Availability

The datasets presented in this study can be found in online repositories. The names of the repository/repositories and accession number(s) can be found below: Stellenbosch University Repository.
